# Harnessing cell reprogramming for cardiac biological pacing

**DOI:** 10.1186/s12929-023-00970-y

**Published:** 2023-08-26

**Authors:** Chih-Min Liu, Yi-Chun Chen, Yu-Feng Hu

**Affiliations:** 1 Division of Cardiology, Department of Medicine, Heart Rhythm Center, Taipei Veterans General Hospital, Taipei, Taiwan; 2 Faculty of Medicine and Institute of Clinical Medicine, National Yang Ming Chiao Tung University, Taipei, Taiwan; 3 Institute of Biomedical Sciences, Academia Sinica, Taipei, Taiwan; 4 Institute of Biopharmaceutical Sciences, College of Pharmaceutical Sciences, National Yang Ming Chiao Tung University, Taipei, Taiwan

**Keywords:** Biological pacemaker, Electronic pacemaker, Reprogramming, Functional re-engineering, Stem cell, Sinoatrial node, Gene transfer, Biomaterial, Silk fibroin, Bradyarrhythmia

## Abstract

Electrical impulses from cardiac pacemaker cardiomyocytes initiate cardiac contraction and blood pumping and maintain life. Abnormal electrical impulses bring patients with low heart rates to cardiac arrest. The current therapy is to implant electronic devices to generate backup electricity. However, complications inherent to electronic devices remain unbearable suffering. Therefore, cardiac biological pacing has been developed as a hardware-free alternative. The approaches to generating biological pacing have evolved recently using cell reprogramming technology to generate pacemaker cardiomyocytes in-vivo or in-vitro. Different from conventional methods by electrical re-engineering, reprogramming-based biological pacing recapitulates various phenotypes of de novo pacemaker cardiomyocytes and is more physiological, efficient, and easy for clinical implementation. This article reviews the present state of the art in reprogramming-based biological pacing. We begin with the rationale for this new approach and review its advances in creating a biological pacemaker to treat bradyarrhythmia.

## Introduction

Pacemaker cardiomyocytes (PCs) within the sinoatrial node (SAN) trigger a periodical electrical automaticity, initiating heartbeats for circulation. Their automaticity is generated by a coupled system of membrane and calcium clocks (Ca^2+^ clock) [1–4]. The membrane clock is a cyclic change of membrane potential on PCs, attributed to the dynamic ion flow through ion channels, including mainly HCNs (hyperpolarization-activated cyclic nucleotide-gated) channels, L- and T-type Ca^2+^ channels, and delayed rectifier potassium channels (I_K1_) [2]. The Ca^2+^ clock is an intracellular Ca^2+^ cycling kinetic, attributed to localized periodic calcium releases via ryanodine receptors on the sarcoplasmic reticulum [5, 6]. Two clocks work synchronously to generate spontaneous action potentials and control the timekeeping mechanism of heart rhythm. These functionally specialized PCs also possess distinct gene expression profiles and transcriptional regulation from embryogenesis to mature tissue to execute spontaneous firing [7–11]. To be noted, transcription factors during embryogenesis drive PC differentiation include Shox2 (short stature homeobox 2) [9], Tbx3 (T-box transcription factor 3) [12], Tbx18 [13], Isl1 (ISL LIM homeobox 1) [14], and a loss of Nkx2.5 (NK2 homeobox 5) [15].

### The evolution from electronic devices to biological pacemakers

**Fig. 1:**
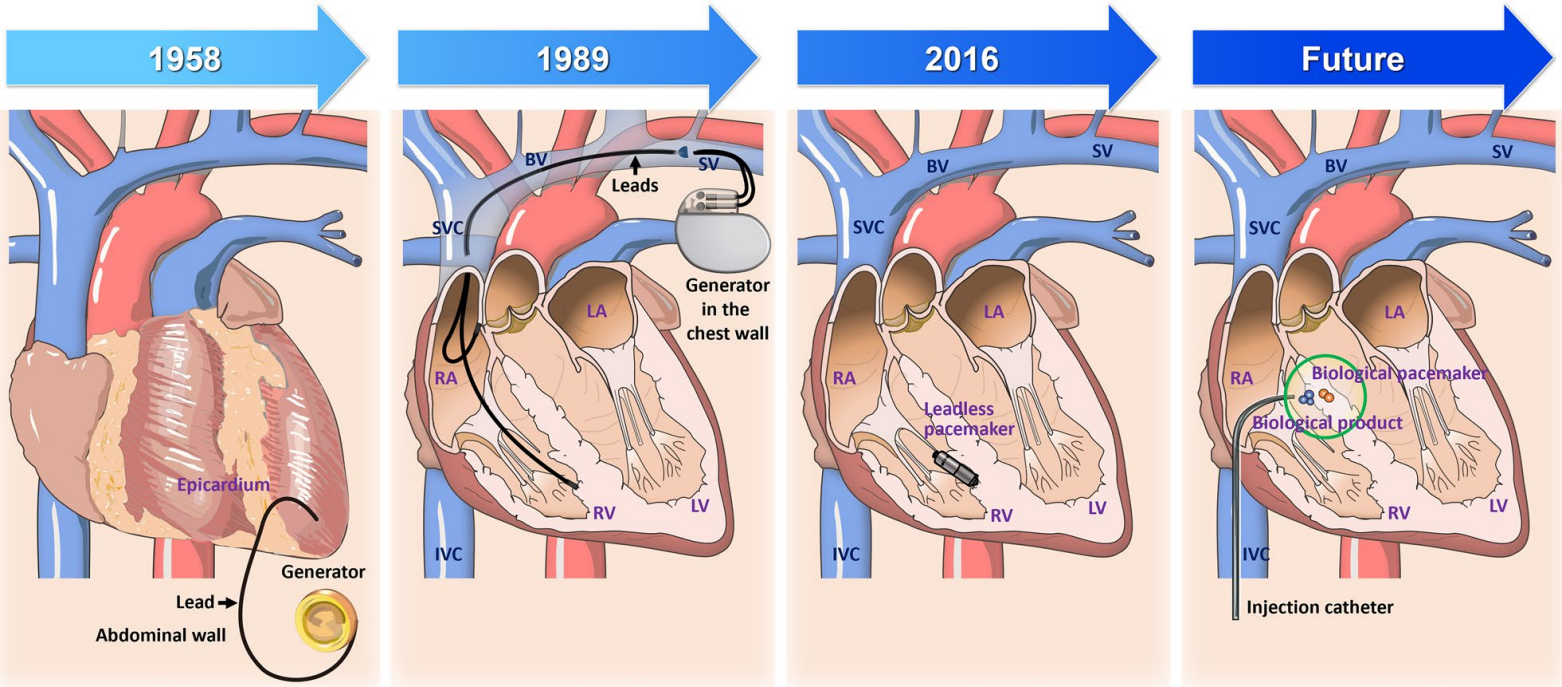
The evolution of cardiac pacing from electronic devices to biological pacemakers. In 1958, the medical world witnessed a breakthrough when the first fully implantable pacemaker was introduced. This used an epicardial lead fixed at the epicardial site of the ventricle. The pacemaker lead was connected to the generator within the generator pocket, which has been formed in the abdominal wall within the rectus abdominus muscle sheath, typically at the level of the umbilicus. Placing epicardial leads and the generator pocket led to lead failure or device infection. Since 1989, the transvenous approach, which inserted the leads through the subclavian veins (SV), replaced epicardial lead implantation as the mainstream procedure. The generator was typically inserted into a pocket just above the pectoral fascia (subcutaneously in the chest wall). Furthermore, the transvenous leads could be fixed through the endocardial site over the right atrium (RA) and ventricle (RV). This made synchronized electrical pacing on atrial and ventricular chambers possible, providing a more physiological way similar to normal atrioventricular conduction. Fast forward to 2016, the Food and Drug Administration approved the marketing of a leadless cardiac pacemaker. This is a one-piece device including a generator and electrodes, implanted into RV septum through a vein. There was neither a separate battery under the skin nor leads that go to the heart. Looking ahead, the dawn of the era of biological pacemakers is on the horizon. The biological pacemaker has been developed as a device-free therapy which injects a biological product (blue dots) through the catheter, e.g., viral vector, to generate a biological pacemaker in situ (orange dots). The biological pacemakers generate a natural and efficient heart rhythm from PCs similar to a de novo sinoatrial node, and might provide physiological pacing compatible with normal cardiac conduction. This further avoids the complications from the electrical pacing of the electronic device, e.g., heart failure. BV, brachiocephalic vein; IVC, inferior vena cava; LA, left atrium; LV, left ventricle; RA, right atrium; RV, right ventricle; SV, subclavian vein; SVC, superior vena cava

SAN dysfunction leads to rhythmic failure and bradycardia. Electric pacemaker implantation to provide backup pacing is an effective and standard therapy. This device includes a pulse generator implanted in the subcutaneous chest space and transvenous/epicardial leads inserted into the atrial or ventricular myocardium (Fig. 1) [16, 17]. The metal case generator contains a battery and a tiny computer to generate and control electrical impulses. The electrical stimulation from the generator is conducted through the insulated wires, called lead, into heart muscles. Since the first fully implantable pacemaker was developed in 1958 at the Karolinska Institute in Sweden, the technology has significantly advanced from mobility-limited devices with short battery life to small generators with batteries lasting 8–10 years [18, 19]. The electronic pacemaker has been a reliable and standard technology for decades. However, complications related to surgical procedures and devices remain evident, including generator or lead malfunctions, infections, lack of physiological autonomic response, and heart failure [20–23].

Scientific exploration keeps searching for ways to reduce the hardware of a pacemaker and eventually, a device-free treatment. A leadless pacemaker has been launched to eliminate complications from the implantation of leads or generators, which inserts the small generator with electrodes directly into the right ventricular septum to avoid lead insertion [24]. The leadless pacemaker avoids the problem of lead implantation and subcutaneous devices, but can only provide single-chamber pacing with limited retrievability. A higher incidence of short-term complications like cardiac perforation or pericarditis has been observed. In parallel, a biological pacemaker has been actively explored because of its potential to avoid device-related complications and physiological compatibility [25]. The strategy by a biological pacemaker is an attractive and device-free treatment in which heartbeats will be generated from biological PCs as same as for genuine human hearts. The evolution from electronic pacemakers to biological pacemakers is described in Fig. 1.

### The developmental strategies of biological pacemakers

**Fig. 2:**
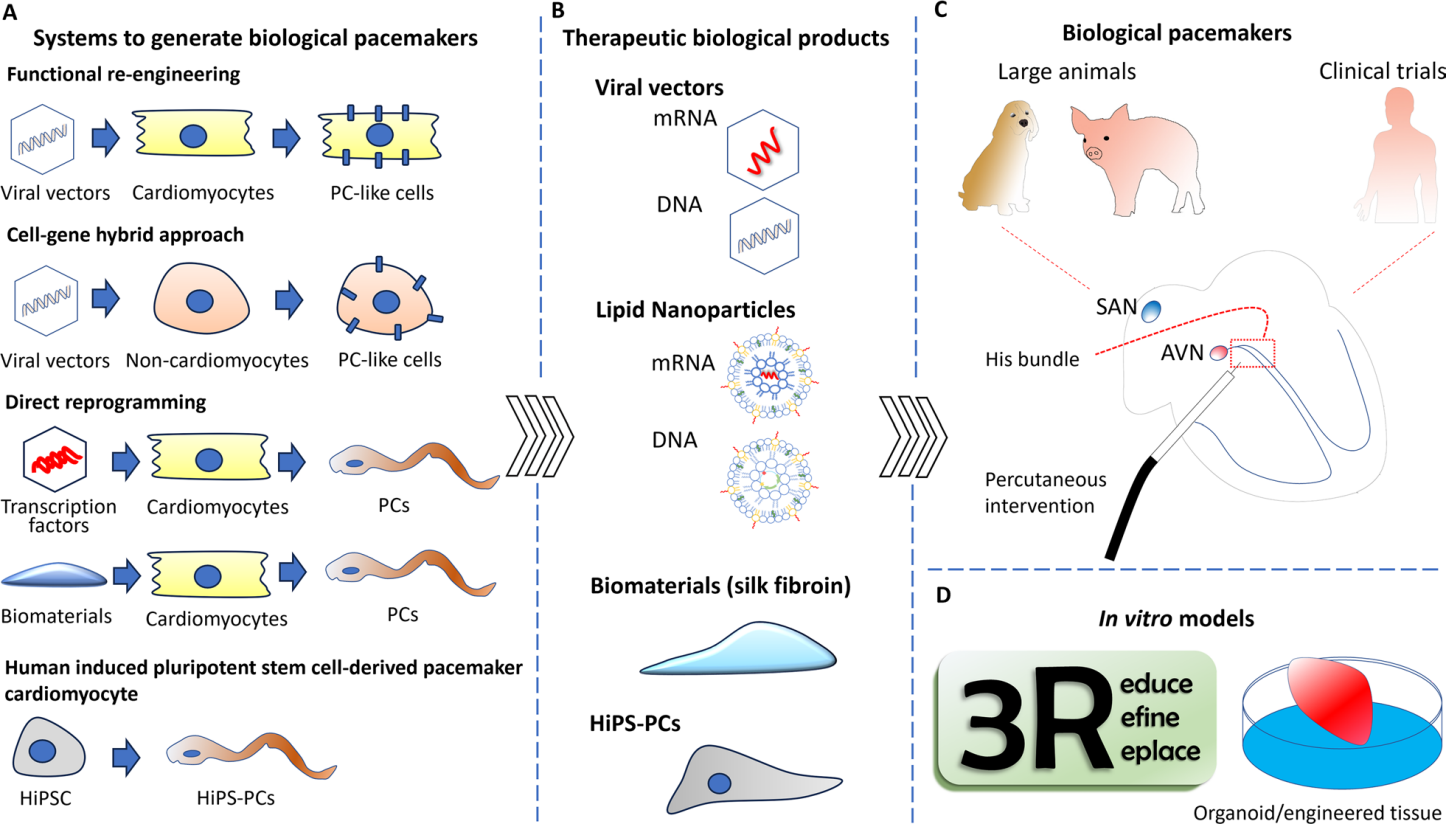
The developmental strategies to generate a biological pacemaker. **A** Different systems to generate biological PCs. Functional re-engineering induces the expression of specific ion channels in VMs to generate ion currents to generate electrical firing. Other than this, cell morphology, structure, and functions remain the same as VMs. The ion channel genes could be expressed in non-cardiomyocyte cells (e.g., MSCs) to generate ion currents to induce pacemaker activity. However, spontaneous action potentials from engineered non-cardiomyocyte cells must be coupled with the nearby VMs. This explains why the efficacy of the cell-gene hybrid approach is worse than those of engineered VMs. Direct reprogramming changes VMs to PCs with holistic changes of morphology, structure, function, and transcriptions. The reprogramming could be reached by the re-expression of transcription factors (e.g., Tbx18) or biomaterials (e.g., silk fibroin). The last is the strategy of cell therapy. Human IPS-PCs could be derived from the subtype differentiation of human IPS cells, and implanted into the heart directly. **B** Biological products for the therapy. The virus (adeno or adeno-associated) or non-viral vectors (lipid nanoparticles for mRNA, DNA, and minicircle DNA) could express the candidate genes for VMs for functional re-engineering or direct reprogramming. The biomaterial per se could be applied as the biological product to induce reprogramming in the heart. For cell therapy, immunocompatible human IPS-PCs could be implanted into the heart with different designs, including PCs alone, cell mixture (e.g., with MSCs), or engineered PC tissues. **C** A minimally invasive procedure should be used to deliver the biological products into the heart from preclinical large animal models to humans. We illustrate that the vectors could be delivered by transvenous catheter into the specific area of the ventricular septum, His bundle, to generate a biological pacemaker. **D** The biological pacemaker could be used to construct organoids (self-organized three-dimensional SANs) or engineered SAN-like tissues. These could be applied as the in-vitro model for screening drugs or exploring pathogenesis, and replace the animals for the preclinical studies. HiPSC, human induced pluripotent stem cell; AVN, atrioventricular node

The biological pacemakers could be generated through different strategies, including functional re-engineering (expressions of specific ion channels), cell-gene hybrid approaches, and direct reprogramming (re-expression of embryonic transcription factors or biomaterial induction) or the transplantation of pacemaker cells derived from human embryonic stem cells (hESC) or induced pluripotent stem cells (iPSC) as detailed in several reviews [26, 27]. An effective biological pacemaker available for clinical translation must deliver a sufficient heart rate, autonomic response, and long-term pacemaker activity [27]. In the present work, we will address the critical issues determining a successful translation of biological pacemakers into clinics, primarily through a reprogramming strategy. These include biological products (gene, vector, or biomaterials), delivery methods, target populations, and longevity/persistence [28]. The strategies and future designs to generate biological pacemakers are summarized in Table 1 and Fig. 2.

**Table 1:** Strategies for developing biological pacemakers

Strategy	Animal/delivery	Beating rate (bpm)	MDP (mV)	APD_90_ (ms)	*I*_f_ (pA/pF)	Decreased genes	Increased genes	No changes	Duration (days)	References
Functional re-engineering										
Adeno-Kir2.1AAA	Guinea pig/LV	300	− 60.7 ± 2.1	–	–	–	–	–	3–4	Miake et al. [36]
Adeno-HCN2	Canine/LA appendage	–	–	–	45.3 ± 12.5 (at − -125 mV)	–	–	–	3–4	Qu et al. [29]
Adeno-HCN2	Canine/LBBB	60	− 65	–	50 (at − 115 mV)	–	–	–	7	Plotnikov et al. [37]
Adeno-HCN2 (E324A)	Canine/LBBB	52	–	–	21.0 ± 3.5 (at − 125 mV)	–	–	–	14	Bucchi et al. [42]
Adeno-HCN2/SkM1	Canine/LB	80	− 85 ± 1	224 ± 9	–	–	–	–	7	Boink et al. [33]
Adeno-SK4/HCN2	Rat/LV	139.9 ± 21.9	–	82.5 ± 12.8	–	–	Kcnn4 Hcn2	–	5–7	Zhao et al. [121]
Cell-gene hybrid approach										
Lenti-HCN1-fibroblasts fused with myocytes	Guinea pig/LV	180	− 54	–	− 54 (at − 125 mV)	–	–	–	22	Cho et al. [40]
pIRSE-HCN2-MSCs	Canine/LVE	60	–	–	–	–	–	–	42	Plotnikov et al. [43]
Lenti-HCN4-MSCs	Canine/LVE	59 ± 5	–	–	26.1 ± 3.6 (at − 140 mV)	–	Hcn4	–	42	Lu et al. [46]
Lenti-HCN2/SkM1-CPCs	Mice/LV	–	–	–	–	–	–	–	7	Végh et al. [47]
Direct reprogramming										
Tbx3 transgenic mice	Mice	32	− 60	164	–	Cx43 Cx40 Scn5a Nppa Kir2.1 Kir3.1 Kir2.2	Hcn1 Lbh	Hcn2 Hcn4	5	Bakker et al. [53]
Adeno-Tbx18	Guinea pig/LV	135	− 37	119		Cx43 Kir2.1 Nav1.5 PLB Nkx2-5 α-actinin	P-PLB	Serca2a Ncx1 Ryr	28	Kapoor et al. [51]
Adeno-Tbx18	Pig/RV septum	58	–	274	–	Nav1.5 Nkx2.5 Kir2.1 Cx43	Hcn4	Cx45 α-actinin	14	Hu et al. [91]
CMmTbx18 with antagomirs	Rat/LV	97	–	–	–	Cx43 SCN5a Nkx2.5	Hcn2 Hcn4 Tbx18		5	Sanchez et al. [58]
Biomaterials (silk fibroin)	Rat/LV	137	− 59	–	18 (at − 150 mV)	Myl2 Cx43	P-PLB Hcn4 Cx45 Cx40	Nkx2.5	56	Hu et al. [64]
Human induced pluripotent stem cell-derived pacemaker cardiomyocytes										
Lenti-Tbx18-hiPSC-CMs	Rat/LV	219 ± 18	–	–	–	Cx43	Tbx18 Hcn4	–	14	Gorabi et al. [122]
HiPSC-CMs	Canine/LV	69 ± 10.4	–	–	− 87 (at − 120 mV)	Nanog Okt-4 Pou5flc12 Pou5flc1 Pou5flc8 Utf1 Nodal Tdgf1	Nkx2.5 Anf Csrp3 Phospholamban Des Myh6 Myh7 Tnnt4	–	91	Chauveau et al. [44]

The relevant data provided in the manuscript is included in the table. If not available, measurements were taken based on the scale of spontaneous action potential provided on the chart or graph

*CMmRNA* chemically modified mRNA, *CPCs* cardiac progenitor cells, *HiPSC-CMs* Human induced pluripotent stem cell-derived cardiomyocytes, *I*_*f*_ funny current, *LA* left atrium, *MSCs* mesenchymal stem cells, *pIRES* internal ribosome entry site containing plasmid, *RV* right ventricle, *LBBB* left bundle branch, *LV* left ventricle, *LVE* left ventricle epicardium, *MDP (mV)* maximum diastolic potential, *APD90* action potential duration at 90% repolarization (ms)

#### Functional re-engineering of genes to generate pacemaker currents

To recapitulate the electrical function of PCs, ectopic expressions of genes related to pacemaker currents (membrane or Ca^2+^ clocks) into quiescent ventricular cardiomyocytes (VMs) generate spontaneous action potentials and electrical firing in these VMs [2]. This is a process referred to as functional re-engineering [29]. The expressions of relevant genes (β2-adrenergic receptor, Kir2.1AAA, HCNs or SkM1[skeletal muscle sodium channel 1], calcium-stimulated adenylyl cyclase [AC1]) [30–34] that are responsible for membrane clock could generate spontaneous action potential and biological pacemaker activity within quiescent atrial or VMs that otherwise remain structurally and genetically unchanged [35]. A mutant gene (mutant Kir2.1AAA or HCNs) or a combination of two genes (HCN2 with Kir2.1AAA or SkM1) was used to change the kinetics of pacemaker current and enhance firing rates [29, 33, 36–38].

The over-expression of β_2_-adrenergic receptors in cardiomyocytes failed to generate de novo automaticity and only enhanced endogenous heart rates [31, 39]. The expression of a mutant Kir2.1 channel by Adenoviral Kir2.1AAA in guinea pig ventricles successfully created spontaneous action potential but was potentially complicated with a proarrhythmic long QT phenotype [36]. The overexpression of genes encoding HCN channels (HCN1, HCN2, and HCN4) through different vectors or cell fusion has been tested in different models, from in-vitro cells and guinea pigs to canines [29, 34, 40, 41]. Although HCNs could induce spontaneous pacemaker activity, their electrical firing rate was neither tested in large animals nor achieved a sustainable or clinically relevant heart rate of 60 to 90 beats/min. Therefore, mutant HCN2 (E324A, HCN212), calcium-stimulated adenylyl cyclase, combo genes (HCN2/AC1, HCN2/Kir2.1AAA, or HCN2/SkM1) were developed to enhance pacemaker activity [33, 38, 42]. Only HCN2/Kir2.1AAA, and HCN2/SkM1 satisfy the basal requirement of heart rates (60–90 bpm), and minimal electrical backup pacing. HCN2/AC1 resulted in an excessive increase in basal beating rate of around 130 bpm. Only HCN2/SkM1 combination achieves clinical requirements within seven days after gene delivery.

#### Cell-gene hybrid approach

The ectopic expressions of genes related to pacemaker currents (membrane or Ca^2+^ clocks) could also be transduced into non-cardiomyocyte cells and generate pacemaker currents [40]. These non-cardiomyocyte cells include human mesenchymal stem cells (hMSCs), human cardiomyocyte progenitor cells (CPCs), and fibroblasts [40, 43, 44]. The transplantation of these engineered non-cardiomyocyte cells could drive the electrical activity of the nearby VMs through cell fusion or electrical coupling between the engineered non-cardiomyocyte cells and VMs [40, 43, 45–47]. This is referred to as a cell-gene hybrid approach. This strategy might benefit their immune privilege, e.g., MSCs [33]. The introduction of HCNs (HCN1, 2, and 4) in those non-cardiomyocyte cells could generate pacemaker ion currents (I*f*) in the in-vitro cell models and pace the heart with bradycardia. HCN2 or HCN4-transduced MSCs required at least 2–3 weeks to fully stabilize and manifest with biological pacemaker activity in dogs with atrioventricular (AV) block [43, 46]. This is probably attributed to the time to form a mature cell–cell junction between MSC or cell–cell fusion [40, 43, 45–47]. This approach was associated with a relatively low heart rate (basal rate of 50–60 bpm) of biological pacemakers and concerns about migration and differentiation of hMSCs [43, 48, 49]. Végh et al. showed that re-engineered cardiac progenitor cells with HCN2/SkM1 gene transduction using nucleofection or lentiviral transduction could generate Hcn2 and Skm1 currents. Low cell engraftment of the engineered cells was observed, and the in-vivo pacemaker features were not reported [47].

#### Direct reprogramming of quiescent ventricular cardiomyocytes to pacemaker cardiomyocytes

The combo-gene strategy overcomes a single gene’s limitation and meets clinically relevant heart rates. This means successful biological PCs might need to be shaped through different mechanisms simultaneously. During embryonic SAN development, Shox2, Tbx3, Tbx5, and Tbx18 transcription factors are spatially and temporally expressed to regulate the differentiation and specification of SAN progenitor cells [50]. Recent works have shown that the re-expression of these transcriptional factors might drive the direct reprogramming of VMs to PCs [51–54]. Direct reprogramming induces a holistic change of cellular structures or electrophysiology. The reprogrammed cells faithfully recapitulate the sophisticated pacemaker cell phenotype, which oscillates electrically and exhibits the fine nuances of calcium clock behavior and distinctive morphological features of genuine SAN pacemakers [51]. The reprogrammed cells are much similar to genuine PCs and, therefore, considered better than functional re-engineering approaches, which created biological pacemakers by manipulating end-effectors of cardiac electrophysiology (membrane or Ca^2+^ clock).

The first trial was done by Bakker et al., who induced Tbx3 expression ectopically in cardiomyocytes of adult transgenic mice using tamoxifen [53]. Tbx3 expression reprogrammed mature VMs into pacemaker-like cells by reducing I_K1_ and intercellular coupling. The downregulation of working myocardial genes such as Cx43, Cx40, Scn5a, Nppa, Kir2.1, Kir3.1, and Kir2.2 was also observed [53]. Kapoor et al. further screened and tested a set of transcription factors during the embryonic stage of PCs. Compared to other transcription factors (Shox2, Tbx3, Tbx5, and Tbx20), human Tbx18, transduced by an adenoviral vector, induced trans-differentiation of adult VMs to PCs. From in-vitro cell model to in-vivo guinea pig’s hearts, Tbx18-transduced VMs acquire morphological phenotypes and physiological automaticity of native SAN pacemaker cells with epigenetic modification of relevant pacemaker genes including Cx43, Kir2, Actc2, and HCN4 [51]. Massive electrogenic, metabolic, and cytostructural remodeling of VMs has been observed, and intracellular cytoskeletal and extracellular matrix remodeling exhibit hallmarks of the epithelial-to-mesenchymal transition [55, 56]. The adenoviral Tbx18 delivery into pigs’ hearts with bradycardia could generate biological pacemakers to support physical activity.

Following these initial successes, several delivery technologies through adeno-associated virus or chemical-modified mRNAs have been developed to induce Tbx18 expression in VMs, but avoid adenoviral vector-related immunogenicity [57, 58]. Tbx18 expression by the adeno-associated viral vector induced reprogramming and generated PCs in the in-vitro cell models [57]. RNA therapeutics should be highlighted as those comprise a rapidly expanding category of drugs for many diseases [59, 60]. The Tbx18 mRNAs were chemically modified to avoid an immune response triggered by naked RNA [58]. However, the Tbx18 expression after the delivery of modified Tbx18 mRNAs was compromised by the activation of miR-1-3p and miR-1b. Therefore, the sustained Tbx18 expressions could only be reached by combining modified Tbx18 mRNAs and antagomirs of miR-1-3p and miR-1b, which induced de novo biological pacemaker activity at the injection site in rats’ hearts [58].

In addition to transcription factors, biomaterials can also stimulate cell reprogramming into PCs. Smith et al. demonstrated direct reprogramming of Yamanaka factors-expressing cultured fibroblasts into cardiomyocyte-like cells on Polyethylene glycol (PEG) hydrogel coated with laminin and arginine-glycine-aspartic acid (RGD) peptides. PEG induces cell reprogramming and conduction gene expression compared to Matrigel-coated polystyrene controls, suggesting that biomaterials may enhance PC reprogramming or generation [61]. The biomaterials strategy has been further advanced by silk fibroin (SF), a natural protein polymer approved for clinical use by the US Food and Drug Administration. The material has been studied and fabricated for tissue engineering and regenerative medicine applications [62, 63]. Hu et al. found that SF per se could reprogram cardiomyocytes to be PCs without any gene delivery [64]. SF induced VMs to transdifferentiate into PCs through the Cdh5 (cadherin 5)/β-catenin pathway. The external mechanical stimuli generated by SF transmitted through surface adhesion molecules could determine cardiac cell fate and maturity during indirect reprogramming [65, 66]. SF-induced PCs have similar electrophysiology and morphology phenotypes and gene expression profiles to native SAN PCs. Furthermore, the SF-induced PCs had prominent autonomic responses and could pace the rat heart with a complete heart block. A translational study using a large-animal model to assess safety and efficacy is necessary. Biomaterials have the advantages of low manufacturing costs and scalable flexibility. This is a vector and gene-free therapy to induce reprogramming; therefore, complications related to gene therapy could be significantly avoided.

#### Human induced pluripotent stem cell-derived pacemaker cardiomyocytes

Chauveau et al. transplanted beating embryoid bodies differentiated human keratinocyte-derived iPSCs to create a biological pacemaker in the left ventricular epicardium of canines for a 3-month follow-up [44]. The rhythms originating from injection sites were present 50 percent of the time and had a relatively low rate of 40 to 50 beats per minute. The injected cells’ retention rate was not reported, but critical for their electrical activity. The integration of iPSCs within donors’ hearts alters the electrical conduction and beating rate [44, 67]. Currently, iPSC technologies result in a mixed population of cells with varying phenotypes, which might affect the function of iPSC-derived biological pacemakers. Reducing immune response from human iPSC by autologous or match cells may enhance the *in-vivo* biological pacemaker function [68]. The iPSC generation is time-consuming (6–12 weeks) and costly [69, 70]. There may be a risk of tumorigenicity associated with the transplantation of iPSC [71]. All of these issues need to be addressed.

#### Vectors for efficient gene expressions

The efficient target gene expression is vital for successfully engineering or reprogramming PCs. Except for biomaterials, viruses (adenovirus or lentivirus) were selected as the only strategy to express the genes within cardiomyocytes because of their better efficacy in all exploratory experiments, as mentioned above [72]. However, these vectors are limited by unfavorable clinical concerns of future applications. Adenoviral vectors are commonly used in gene therapy due to their large packaging capacities and transient gene expression. However, their immunogenicity and cellular toxicity present significant obstacles to long-term applications [73]. Lentiviral vector also allows for constructing a transgene up to 10 kb. It preferentially targets the transcriptional unit of the host genome, allowing for highly abundant and long-term multiple transgene expression [74–76]. However, insertional mutagenesis and oncogenesis remain critical for its clinical use in the heart [77]. Recombinant adeno-associated virus is one of the alternative favorable vectors to avoid immunogenicity, cytotoxicity, and mutagenesis. However, the limited transgene size might need to be revised to allow their application [78, 79].

Non-viral vectors, including polymers, lipids, inorganic nanoparticles, and peptides, encapsulate DNAs or RNAs, protect these cargos from extra- and intracellular enzymatic digestion, and enhance the efficacy of gene expression [80, 81]. The mRNA-based gene therapy is one of the future stars. The mRNA-based treatment with lipid nanoparticles (LNPs) has improved the expressive efficacy of the gene of interest and reduced immune response by the FDA for mRNA vaccine delivery [82–85]. For precise expression of target genes in the target myocardium, selecting a feasible promoter, such as the cardiac Troponin T promoter, is usually used to reduce the off-target effect [86]. Now, it is also possible for tissue-specific mRNA delivery [87]. LNPs are formulated with four fundamental components: ionized cationic lipids, helper lipids, PEG lipids, and cholesterol [88]. Cheng et al. showed that including a permanently cationic lipid (DOTAP) into LNP compositions could tune the internal charge by which LNP can specifically be delivered into the lung, spleen, or liver [87]. Meanwhile, cardiac distribution of the report genes could also be observed using DOTAP at 15% in LNPs. This suggests that a potential permanently cationic lipid with titration of its molar compositions within LNPs could be designed for cardiac tropism, especially for cardiomyocytes. The minicircle DNAs are also a potential alternative because they have been shown to have better gene expression and duration than bacterial DNA plasmids [89, 90].

### Biological pacemakers from benches to large animals

It is challenging to induce bradycardia in small animals. Therefore, a biological pacemaker's in-vivo electrophysiological functional phenotypes are usually performed in large animals, from canines to pigs. Both are also translational models for the pre-approval of administrative regulation. We summarize the large animal studies in Table 2. The therapy by Tbx18 or HCN2/SkM1 gene delivery achieved much closer to the clinical application: a physiologically biological pacemaker (basal beating rates around 70–90 beats) with a rapid autonomic response in large animals [33, 35, 91]. The procedure could be minimally invasive through the femoral sheath without thoracotomy [91]. Several physiological advantages of biological pacemakers are also suggested. A biological pacemaker could respond appropriately to natural arousal stimuli (triggered by food), exercise, or diurnal changes [91, 92]. Also, autonomic responses are well noted [91]. Notably, normal QT interval and no signs of proarrhythmic or systemic adverse effects were observed during the two-week follow-up in the pig model with Tbx18 [91]. The biological pacemaker activity in the large animal could be observed from 7 days to 6 weeks. A hybrid approach with HCN2 or HCN4 with MSC might sustain more than six weeks. However, the onset takes two weeks [43, 46]. Catheter-delivery of Tbx18 to a specific area of the His-bundle area restored antegrade conduction with biological pacing and prevented electric pacing-induced cardiomyopathy [93]. Human iPSC-derived PCs are an alternative strategy to overcome cell aging or dysfunction within diseased hearts, as these injected cells could be derived from a highly selected healthy population [94].

**Table 2:** Studies involving biological pacemakers in large animals

Studies	Year	Mechanism	Biological agents	Delivery methods	Target animals (injection site)	Functional duration	Monitor device	Backup electronic pacemakers
Functional re-engineering								
Edelberg et al. [39]	2001	β_2_-adrenergic receptors	Plasmid pBR322-β actin promoter-β_2_ adrenergic receptor	Transvenous	Healthy swine (RA)	4 days	Repeated ECGs	No
Qu et al. [29]	2003	HCN2	AdGFP-HCN2	Thoracotomy	Healthy canine (LAA)	4 days	Continuous ECGs on day 4	No
Plotnikov et al. [37]	2004	HCN2	AdGFP-HCN2	Transarterial	Healthy canine (LB)	7 days	Continuous 24-h Holter	No
Bucchi et al. [42]	2006	HCN2	AdGFP-HCN2 AdGFP-E324A	Transarterial	Canine with CAVB (LB)	14 days	Continuous 24-h Holter	Yes
Tse et al. [96]	2006	HCN1	AdGFP-HCN1Delta	Thoracotomy	Swine with SSS (LAA)	14 days	Repeated ECGs	Yes
Plotnikov et al. [123]	2008	HCN2	AdGFP-HCN212	Transarterial	Canine with CAVB (LBB)	14 days	Continuous 24-h Holter	Yes
Shlapakova et al. [92]	2010	HCN2	AdGFP-HCN2	Transarterial	Canine with CAVB (LBB)	7 days	24-h Holter at day 1 and day 7	Yes
Cingolani et al. [38]	2012	HCN2 and Kir2	AdGFP-HCN2 + Kir2.1AAA	Transvenous	Swine with CAVB (AVJ)	14 days	Intermittent 24-h Holter on day 7 and day 14	Yes
Boink et al. [33]	2013	HCN2	AdGFP-HCN2/SkM1 AdGFP-HCN2 AdGFP-SkM1	Transarterial	Canine with CAVB (LB, LV)	7 days	Intermittent 24-h Holter from day 5 to day 7	Yes
Cell-gene hybrid approach								
Potapova et al. [95]	2004	hMSC-HCN2	HCN2-expressing hMSCs	Thoracotomy	Healthy canine (LV)	10 days	Repeated ECGs	No
Plotnikov et al. [43]	2007	hMSC-HCN2	HCN2-expressing hMSCs	Thoracotomy	Canine with CAVB (LV)	6 weeks	24-h Holter weekly	Yes
Direct reprogramming								
Hu et al. [91]	2014	Tbx18	AdGFP-Tbx18	Transvenous	Swine with CAVB (RV septum)	14 days	Real-time, continuous ECG telemetry	Yes
Human induced pluripotent stem cell-derived pacemaker cardiomyocytes								
Chauveau et al. [44]	2017	iPSC	iPSC-derived embryonic body	Thoracotomy	Canine with CAVB (Epicardium of LV)	3 months	24-h Holter biweekly	Yes

*AdGFP* adenoviral construct, and green fluorescent protein, *AVJ* AV junction, *CAVB* complete AV block, *CM* cardiomyocyte, *ECG* electrograms, *hESC* human embryonic stem cells, *hMSCs* human mesenchymal stem cells, *iPSC* induced pluripotent stem cells, *LAA* left atrial appendage, *LB* left bundle, *LV* left ventricle, *RA* right atrium, *RV* right ventricle, *SSS* sick sinus syndrome

### Current limitations and future perspectives in clinical translation

The preclinical data provide robust evidence for the effectiveness of biological pacemakers and reveal some potential limitations to overcome. First, it is imperative that the procedure for delivering a biological pacemaker to humans needs to be as less invasive as possible. Delivery methods in the past involved highly invasive procedures (transarterial or thoracotomy), which limits the potential for translation into humans [37, 42, 95, 96]. In a swine model of complete heart block, Cingolani and Hu et al. have demonstrated that biological pacemakers can be delivered through a catheter inserted into the venous system with minimally invasive techniques [38, 91]. Even so, electro-anatomical mapping and fluoroscopy in the study were used to guide the injection of biological pacemaker constructs. The real-time monitoring of precise injection within the myocardium and reducing leakage is necessary but has not been demonstrated until now. The systemic spreading of vectors could be a problem after the needle injection. Biomaterial might overcome this. SF hydrogel has a higher viscosity and, therefore, will be retained within the injection site without systemic distribution [64]. Second, more work must be done to ensure physiological, immediate-to-use, and durable pacemaker function. Physiologically effective biological pacemaker activity has been observed to reduce electronic backup pacing to minimal usage. However, the activity could be more sustainable [28, 33, 91]. Noteworthy, the studies only proved that the biological pacemaker adjunct to electronic pacemaker could substantially reduce pacing but not alternative to electronic pacemakers in a hardware-free animal model [33, 43, 44, 91]. This will be absolutely needed for a future clinical indication as the alternative to the electronic device. Third, the potential immunogenicity of bioactive agents, including vectors or cells, remains a concern [68, 97]. This might decrease in-vivo gene expressions and the durability of a biological pacemaker. A low immunogenicity vector or optimal immunosuppression strategy after the delivery of vectors or cells needs to be developed.

Overall, the reprogramming strategy might be a better one as PCs recapitulate the de-novo phenotypes of a SAN (Fig. 2). This could be achieved by viral vectors, RNAs, or biomaterials. The conventional approaches need to test the novel treatment from small animals (rats or guinea pigs) to large animals (pigs or canines), eventually humans. The creation of a bradycardia model and real-time monitoring is challenging in small animals, and therefore, functional validation needs to be performed in large animals currently. The development of a small animal model will be a cost-reduction strategy [98]. In addition, concerning animal welfare, an in-vitro organoid system will be necessary to reduce the use of animals, and the best one will be SAN organoids by human iPSC PCs. The delivery method is suggested minimally invasive percutaneous catheter delivery with real-time visual monitoring of location and size.

### Potential clinical niches of biological pacemakers

It remains in the infancy stage for a biological pacemaker to replace the electronic device. However, biological pacemakers might provide a therapeutic alternative for patients with device-related complications or technical difficulty and failure. Currently, available electronic devices have limitations related to lead or generator malfunction, insufficient autonomic response, unfavorable interactions with magnetic fields, and infections [16, 17, 28]. A biological pacemaker may be appropriate in these circumstances. A minimally invasive delivery system is advantageous when delivering the biological pacemaker product for the first-in-human application.

#### Temporal pacing

Even the durability of biological pacemakers is shorter than two weeks. Nevertheless, it remains a perfect indication for replacing temporary electronic pacemakers. Temporary pacemaker wires are prone to loss of capture, under-sensing over time, and restricting patient mobility. Furthermore, using temporary pacing leads before the implantation of a permanent pacemaker is positively correlated with a higher risk of infection [99]. In this regard, a hardware-free, temporary pacing alternative with an effective biological pacemaker may be able to provide temporary pacing.

#### Device-associated infections

Approximately 2% of patients with pacemakers or cardioverter-defibrillators have an infection following their implantation [35, 100]. Patients with transvenous device-related infections have a significantly higher mortality rate (8.4% with pacemaker-related infections), and intensive care accounted for almost half of the incremental admission costs [101]. When another pacemaker is implanted, the possibility of reinfection varies between 2 and 11%, depending on whether the entire system is removed [102]. The biological pacemaker needs no hardware and prevents recurrent infection.

#### No central venous routes for electronic pacemaker

Venous stenosis following transvenous lead implantation ranges from 25 to 64% [103, 104]. Up to 26% of patients require pacemaker revision following the initial implant after 6.2 years [103]. In cases of lack of venous access or occluded veins at the upper extremities, surgical epicardial leads and intracardiac leadless pacemakers are recommended therapeutic alternatives [17]. Transcatheter biological pacemakers, similar to a leadless pacemaker [105], could be a less invasive alternative than the epicardial approach when central vascular access is difficult or unavailable.

#### High surgical risk

A large prospective multicenter study indicates a 10.1% incidence of in-hospital events after the first transvenous pacemaker implantation, such as pneumothorax, cardiac perforation, lead-related events, device-related events, and mortality [106]. Several high-risk factors have been identified for complications following pacemaker implantation in previous studies, such as elderly patients, low body mass indexes, history of heart failure, or dialysis patients [106–108]. Biological pacemakers can be administered using minimally invasive procedures through venous catheter approaches for patients at high surgical risk to reduce complications.

#### Short life expectancy

Deactivating cardiac pacemakers in end-of-life patients is a controversial ethical issue [109, 110]. Approximately 42% of deaths occurred within one day of deactivation, with the median survival time being two days [111]. Palliative patients are less likely to undergo surgical interventions such as intubation, cardiopulmonary resuscitation, or pacemaker implantation. In this circumstance, a minimally invasive procedure with a venous catheter approach to administering a biological pacemaker would be a choice.

#### Permanent atrial fibrillation with slow ventricular response

Patients with permanent atrial fibrillation (AF) associated with AV nodal disease may experience fatigue, dizziness, and syncope symptoms [112]. In these patients, AV synchronization is unnecessary; therefore, single-chamber pacemakers are often used. A minimally invasive biological pacemaker, like a leadless pacemaker [113], would have the advantages of preventing pocket infections, hematomas, lead dislodgment/fractures, and cosmetic appeal compared to the traditional transvenous pacemaker. Nevertheless, the current leadless pacemaker uses an individually programmable three-axis accelerometer (Micra Transcatheter Pacing System). The motion vectors of leadless pacemakers could only achieve an adequate quality of rate adaptive pacing in 74.5% of the patients during the exercise tests [114]. There is a potential for autonomic responsive biological pacemakers to solve the problem and provide a viable alternative to leadless and transvenous pacemakers.

#### Pediatric patients with indications for electronic pacemaker

Approximately one in 20,000 live-born infants suffer from congenital complete AV block, associated with high mortality of up to 34% [115, 116]. A pacemaker is implanted in about 90 percent or more of these patients [117]. Due to the smaller diameter of transvenous insertion veins in infants and very young children and the expectation for thoracic growth causing tension on leads, epicardial leads are typically required [118, 119]. However, epicardial leads are more likely to fracture and experience exit block, which requires significant surgery with all the associated risks for perioperative care [120]. The use of an automatic biological pacemaker may be helpful in pediatric patients with congenital complete AV block since there is no body size limitation and no need for lead revision as the patient grows.

Overall, the advent of minimally invasive biological pacemakers might create a safe and feasible alternative to transvenous lead pacemakers, especially in high-surgical risk patients, pediatric patients, permanent AF patients, palliative patients, or those with vascular access site issues or infections that preclude implantation of an electronic pacemaker.

## Conclusions

Reprogramming strategies advance the development of a biological pacemaker to a brand-new page. Now, induced biological pacemakers are closer to a biological twin to the de-novo SAN. It is optimistically believed that the hurdles, e.g., durability, will eventually be overcome by accumulating efforts before the clinical translation.

## Data Availability

Not applicable.

## Abbreviations

AF: Atrial fibrillation
AV: Atrioventricular
Cdh5: Cadherin 5
CPCs: Cardiomyocyte progenitor cells
HCN: Hyperpolarization-activated cyclic nucleotide-gated
hESCs: Human embryonic stem cells
hMSCs: Human mesenchymal stem cells
I*f*: Pacemaker ion currents
I_K1_: Delayed rectifier potassium channels
iPSC: Induced pluripotent stem cells
LNPs: Lipid nanoparticles
Isl1: ISL LIM homeobox 1
Ncx1: Sodium-calcium exchanger 1
Nkx2.5: NK2 homeobox 5
PCs: Pacemaker cardiomyocytes
PEG: Polyethylene glycol
RGD: Arginine-glycine-aspartic acid
RyR2: Ryanodine receptor 2
SAN: Sinoatrial node
Serca2: Sarco/endoplasmic reticulum Ca^2+^-ATPase 2 pump
Shox2: Short stature homeobox 2
SF: Silk fibroin
SkM1: Skeletal muscle sodium channel 1
SORT: Selective organ targeting
Tbx3: T-box transcription factor 3

## References

[1] Irisawa H, Brown HF, Giles W (1993). Cardiac pacemaking in the sinoatrial node. Physiol Rev.

[2] Baruscotti M, Barbuti A, Bucchi A (2010). The cardiac pacemaker current. J Mol Cell Cardiol.

[3] Mangoni ME, Nargeot J (2008). Genesis and regulation of the heart automaticity. Physiol Rev.

[4] Lakatta EG, Maltsev VA, Vinogradova TM (2010). A coupled SYSTEM of intracellular Ca^2+^ clocks and surface membrane voltage clocks controls the timekeeping mechanism of the heart's pacemaker. Circ Res.

[5] Vinogradova TM, Zhou YY, Maltsev V, Lyashkov A, Stern M, Lakatta EG (2004). Rhythmic ryanodine receptor Ca^2+^ releases during diastolic depolarization of sinoatrial pacemaker cells do not require membrane depolarization. Circ Res.

[6] Lakatta EG, DiFrancesco D (2009). What keeps us ticking: a funny current, a calcium clock, or both?. J Mol Cell Cardiol.

[7] Hoogaars WM, Tessari A, Moorman AF, de Boer PA, Hagoort J, Soufan AT, Campione M, Christoffels VM (2004). The transcriptional repressor Tbx3 delineates the developing central conduction system of the heart. Cardiovasc Res.

[8] Christoffels VM, Mommersteeg MT, Trowe MO, Prall OW, de Gier-de VC, Soufan AT, Bussen M, Schuster-Gossler K, Harvey RP, Moorman AF (2006). Formation of the venous pole of the heart from an Nkx2-5-negative precursor population requires Tbx18. Circ Res.

[9] Blaschke RJ, Hahurij ND, Kuijper S, Just S, Wisse LJ, Deissler K, Maxelon T, Anastassiadis K, Spitzer J, Hardt SE (2007). Targeted mutation reveals essential functions of the homeodomain transcription factor Shox2 in sinoatrial and pacemaking development. Circulation.

[10] Sun Y, Liang X, Najafi N, Cass M, Lin L, Cai CL, Chen J, Evans SM (2007). Islet 1 is expressed in distinct cardiovascular lineages, including pacemaker and coronary vascular cells. Dev Biol.

[11] Weinberger F, Mehrkens D, Friedrich FW, Stubbendorff M, Hua X, Muller JC, Schrepfer S, Evans SM, Carrier L, Eschenhagen T (2012). Localization of Islet-1-positive cells in the healthy and infarcted adult murine heart. Circ Res.

[12] Hoogaars WM, Engel A, Brons JF, Verkerk AO, de Lange FJ, Wong LY, Bakker ML, Clout DE, Wakker V, Barnett P (2007). Tbx3 controls the sinoatrial node gene program and imposes pacemaker function on the atria. Genes Dev.

[13] Wiese C, Grieskamp T, Airik R, Mommersteeg MT, Gardiwal A, de Gier-de VC, Schuster-Gossler K, Moorman AF, Kispert A, Christoffels VM (2009). Formation of the sinus node head and differentiation of sinus node myocardium are independently regulated by Tbx18 and Tbx3. Circ Res.

[14] Liang X, Zhang Q, Cattaneo P, Zhuang S, Gong X, Spann NJ, Jiang C, Cao X, Zhao X, Zhang X (2015). Transcription factor ISL1 is essential for pacemaker development and function. J Clin Invest.

[15] Benson DW, Silberbach GM, Kavanaugh-McHugh A, Cottrill C, Zhang Y, Riggs S, Smalls O, Johnson MC, Watson MS, Seidman JG (1999). Mutations in the cardiac transcription factor NKX2.5 affect diverse cardiac developmental pathways. J Clin Invest.

[16] Kusumoto FM, Schoenfeld MH, Barrett C, Edgerton JR, Ellenbogen KA, Gold MR, Goldschlager NF, Hamilton RM, Joglar JA, Kim RJ (2019). 2018 ACC/AHA/HRS guideline on the evaluation and management of patients with bradycardia and cardiac conduction delay: a report of the American college of cardiology/American heart association task force on clinical practice guidelines and the heart rhythm society. Circulation.

[17] Glikson M, Nielsen JC, Kronborg MB, Michowitz Y, Auricchio A, Barbash IM, Barrabés JA, Boriani G, Braunschweig F, Brignole M (2021). 2021 ESC Guidelines on cardiac pacing and cardiac resynchronization therapy. Eur Heart J.

[18] van Hemel NM, van der Wall EE (2008). 8 October 1958, D Day for the implantable pacemaker. Neth Heart J.

[19] Mond HG, Freitag G (2014). The cardiac implantable electronic device power source: evolution and revolution. Pacing Clin Electrophysiol PACE.

[20] Hauser RG, Hayes DL, Kallinen LM, Cannom DS, Epstein AE, Almquist AK, Song SL, Tyers GF, Vlay SC, Irwin M (2007). Clinical experience with pacemaker pulse generators and transvenous leads: an 8-year prospective multicenter study. Heart Rhythm.

[21] Sohail MR, Uslan DZ, Khan AH, Friedman PA, Hayes DL, Wilson WR, Steckelberg JM, Stoner S, Baddour LM (2007). Management and outcome of permanent pacemaker and implantable cardioverter-defibrillator infections. J Am Coll Cardiol.

[22] Trohman RG, Huang HD, Larsen T, Krishnan K, Sharma PS (2020). Sensors for rate-adaptive pacing: how they work, strengths, and limitations. J Cardiovasc Electrophysiol.

[23] Sweeney MO, Hellkamp AS, Ellenbogen KA, Greenspon AJ, Freedman RA, Lee KL, Lamas GA (2003). Adverse effect of ventricular pacing on heart failure and atrial fibrillation among patients with normal baseline QRS duration in a clinical trial of pacemaker therapy for sinus node dysfunction. Circulation.

[24] Chan KH, McGrady M, Wilcox I (2016). A leadless intracardiac transcatheter pacing system. N Engl J Med.

[25] Rosen MR, Brink PR, Cohen IS, Robinson RB (2008). Cardiac pacing: from biological to electronic … to biological?. Circ Arrhythm Electrophysiol.

[26] Naumova N, Iop L (2021). Bioengineering the cardiac conduction system: advances in cellular, gene, and tissue engineering for heart rhythm regeneration. Front Bioeng Biotechnol..

[27] Komosa ER, Wolfson DW, Bressan M, Cho HC, Ogle BM (2021). Implementing biological pacemakers: design criteria for successful. Circul Arrhyth Electrophysiol..

[28] Cingolani E, Goldhaber JI, Marbán E (2018). Next-generation pacemakers: from small devices to biological pacemakers. Nat Rev Cardiol.

[29] Qu J, Plotnikov AN, Danilo P, Shlapakova I, Cohen IS, Robinson RB, Rosen MR (2003). Expression and function of a biological pacemaker in canine heart. Circulation.

[30] Kryukova YN, Protas L, Robinson RB (2012). Ca^2+^-activated adenylyl cyclase 1 introduces Ca^2+^-dependence to beta-adrenergic stimulation of HCN2 current. J Mol Cell Cardiol.

[31] Edelberg JM, Aird WC, Rosenberg RD (1998). Enhancement of murine cardiac chronotropy by the molecular transfer of the human beta2 adrenergic receptor cDNA. J Clin Invest.

[32] Miake J, Marbán E, Nuss HB (2003). Functional role of inward rectifier current in heart probed by Kir2.1 overexpression and dominant-negative suppression. J Clin Invest.

[33] Boink GJ, Duan L, Nearing BD, Shlapakova IN, Sosunov EA, Anyukhovsky EP, Bobkov E, Kryukova Y, Ozgen N, Danilo P (2013). HCN2/SkM1 gene transfer into canine left bundle branch induces stable, autonomically responsive biological pacing at physiological heart rates. J Am Coll Cardiol.

[34] Boink GJ, Verkerk AO, van Amersfoorth SC, Tasseron SJ, van der Rijt R, Bakker D, Linnenbank AC, van der Meulen J, de Bakker JM, Seppen J (2008). Engineering physiologically controlled pacemaker cells with lentiviral HCN4 gene transfer. J Gene Med.

[35] Rosen MR (2014). Gene therapy and biological pacing. N Engl J Med.

[36] Miake J, Marbán E, Nuss HB (2002). Biological pacemaker created by gene transfer. Nature.

[37] Plotnikov AN, Sosunov EA, Qu J, Shlapakova IN, Anyukhovsky EP, Liu L, Janse MJ, Brink PR, Cohen IS, Robinson RB (2004). Biological pacemaker implanted in canine left bundle branch provides ventricular escape rhythms that have physiologically acceptable rates. Circulation.

[38] Cingolani E, Yee K, Shehata M, Chugh SS, Marbán E, Cho HC (2012). Biological pacemaker created by percutaneous gene delivery via venous catheters in a porcine model of complete heart block. Heart Rhythm.

[39] Edelberg JM, Huang DT, Josephson ME, Rosenberg RD (2001). Molecular enhancement of porcine cardiac chronotropy. Heart (British Cardiac Society).

[40] Cho HC, Kashiwakura Y, Marbán E (2007). Creation of a biological pacemaker by cell fusion. Circ Res.

[41] Qu J, Barbuti A, Protas L, Santoro B, Cohen IS, Robinson RB (2001). HCN2 overexpression in newborn and adult ventricular myocytes: distinct effects on gating and excitability. Circ Res.

[42] Bucchi A, Plotnikov AN, Shlapakova I, Danilo P, Kryukova Y, Qu J, Lu Z, Liu H, Pan Z, Potapova I (2006). Wild-type and mutant HCN channels in a tandem biological-electronic cardiac pacemaker. Circulation.

[43] Plotnikov AN, Shlapakova I, Szabolcs MJ, Danilo P, Lorell BH, Potapova IA, Lu Z, Rosen AB, Mathias RT, Brink PR (2007). Xenografted adult human mesenchymal stem cells provide a platform for sustained biological pacemaker function in canine heart. Circulation.

[44] Chauveau S, Anyukhovsky EP, Ben-Ari M, Naor S, Jiang YP, Danilo P, Rahim T, Burke S, Qiu X, Potapova IA (2017). Induced pluripotent stem cell-derived cardiomyocytes provide in vivo biological pacemaker function. Circul Arrhyth Electrophysiol..

[45] Tong S, Yao Q, Wan Y, Zhou J, Shu M, Zhong L, Li Y, Zhang Q, Yindai J, Song Z (2009). Development of functional I f channels in mMSCs after transfection with mHCN4: effects on cell morphology and mechanical activity in vitro. Cardiology.

[46] Lu W, Yaoming N, Boli R, Jun C, Changhai Z, Yang Z, Zhiyuan S (2013). mHCN4 genetically modified canine mesenchymal stem cells provide biological pacemaking function in complete dogs with atrioventricular block. Pacing Clin Electrophysiol.

[47] Vegh AMD, Verkerk AO, Cocera Ortega L, Wang J, Geerts D, Klerk M, Lodder K, Nobel R, Tijsen AJ, Devalla HD (2020). Toward biological pacing by cellular delivery of Hcn2/SkM1. Front Physiol.

[48] Boink GJ, Christoffels VM, Robinson RB, Tan HL (2015). The past, present, and future of pacemaker therapies. Trends Cardiovasc Med.

[49] Liechty KW, MacKenzie TC, Shaaban AF, Radu A, Moseley AM, Deans R, Marshak DR, Flake AW (2000). Human mesenchymal stem cells engraft and demonstrate site-specific differentiation after in utero transplantation in sheep. Nat Med.

[50] Raghunathan S, Islas JF, Mistretta B, Iyer D, Shi L, Gunaratne PH, Ko G, Schwartz RJ, McConnell BK (2020). Conversion of human cardiac progenitor cells into cardiac pacemaker-like cells. J Mol Cell Cardiol.

[51] Kapoor N, Liang W, Marbán E, Cho HC (2013). Direct conversion of quiescent cardiomyocytes to pacemaker cells by expression of Tbx18. Nat Biotechnol.

[52] Kapoor N, Galang G, Marbán E, Cho HC (2011). Transcriptional suppression of connexin43 by TBX18 undermines cell-cell electrical coupling in postnatal cardiomyocytes. J Biol Chem.

[53] Bakker ML, Boink GJ, Boukens BJ, Verkerk AO, van den Boogaard M, den Haan AD, Hoogaars WM, Buermans HP, de Bakker JM, Seppen J (2012). T-box transcription factor TBX3 reprogrammes mature cardiac myocytes into pacemaker-like cells. Cardiovasc Res.

[54] Frank DU, Carter KL, Thomas KR, Burr RM, Bakker ML, Coetzee WA, Tristani-Firouzi M, Bamshad MJ, Christoffels VM, Moon AM (2012). Lethal arrhythmias in Tbx3-deficient mice reveal extreme dosage sensitivity of cardiac conduction system function and homeostasis. Proc Natl Acad Sci USA.

[55] Foster DB, Gu JM, Kim EH, Wolfson DW, O'Meally R, Cole RN, Cho HC (2022). Tbx18 orchestrates cytostructural transdifferentiation of cardiomyocytes to pacemaker cells by recruiting the epithelial-mesenchymal transition program. J Proteome Res.

[56] Chou PC, Liu CM, Weng CH, Yang KC, Cheng ML, Lin YC, Yang RB, Shyu BC, Shyue SK, Liu JD (2022). Fibroblasts drive metabolic reprogramming in pacemaker cardiomyocytes. Circ Res.

[57] Farraha M, Rao R, Igoor S, Le TYL, Barry MA, Davey C, Kok C, Chong JJH, Kizana E (2022). Recombinant adeno-associated viral vector-mediated gene transfer of hTBX18 generates pacemaker cells from ventricular cardiomyocytes. Int J Mol Sci.

[58] Sanchez L, Mesquita T, Zhang R, Liao K, Rogers R, Lin YN, Miguel-Dos-Santos R, Akhmerov A, Li L, Nawaz A (2022). MicroRNA-dependent suppression of biological pacemaker activity induced by TBX18. Cell Rep Med..

[59] Zhu Y, Zhu L, Wang X, Jin H (2022). RNA-based therapeutics: an overview and prospectus. Cell Death Dis.

[60] Kim YK (2022). RNA therapy: rich history, various applications and unlimited future prospects. Exp Mol Med.

[61] Smith AW, Hoyne JD, Nguyen PK, McCreedy DA, Aly H, Efimov IR, Rentschler S, Elbert DL (2013). Direct reprogramming of mouse fibroblasts to cardiomyocyte-like cells using Yamanaka factors on engineered poly(ethylene glycol) (PEG) hydrogels. Biomaterials.

[62] Kambe Y, Yamaoka T (2019). Biodegradation of injectable silk fibroin hydrogel prevents negative left ventricular remodeling after myocardial infarction. Biomater Sci.

[63] Kambe Y (2021). Functionalization of silk fibroin-based biomaterials for tissue engineering. Polym J.

[64] Hu YF, Lee AS, Chang SL, Lin SF, Weng CH, Lo HY, Chou PC, Tsai YN, Sung YL, Chen CC (2022). Biomaterial-induced conversion of quiescent cardiomyocytes into pacemaker cells in rats. Nat Biomed Eng.

[65] Paoletti C, Divieto C, Chiono V (2018). Impact of biomaterials on differentiation and reprogramming approaches for the generation of functional cardiomyocytes. Cells.

[66] Kong YP, Rioja AY, Xue X, Sun Y, Fu J, Putnam AJ (2018). A systems mechanobiology model to predict cardiac reprogramming outcomes on different biomaterials. Biomaterials.

[67] Protze SI, Liu J, Nussinovitch U, Ohana L, Backx PH, Gepstein L, Keller GM (2017). Sinoatrial node cardiomyocytes derived from human pluripotent cells function as a biological pacemaker. Nat Biotechnol.

[68] Zhao T, Zhang ZN, Rong Z, Xu Y (2011). Immunogenicity of induced pluripotent stem cells. Nature.

[69] Rhee JW, Wu JC (2018). Cardiac cell cycle activation as a strategy to improve iPSC-derived cardiomyocyte therapy. Circ Res.

[70] Polo JM, Liu S, Figueroa ME, Kulalert W, Eminli S, Tan KY, Apostolou E, Stadtfeld M, Li Y, Shioda T (2010). Cell type of origin influences the molecular and functional properties of mouse induced pluripotent stem cells. Nat Biotechnol.

[71] Lee AS, Tang C, Rao MS, Weissman IL, Wu JC (2013). Tumorigenicity as a clinical hurdle for pluripotent stem cell therapies. Nat Med.

[72] Merentie M, Lottonen-Raikaslehto L, Parviainen V, Huusko J, Pikkarainen S, Mendel M, Laham-Karam N, Kärjä V, Rissanen R, Hedman M (2016). Efficacy and safety of myocardial gene transfer of adenovirus, adeno-associated virus and lentivirus vectors in the mouse heart. Gene Ther.

[73] Bulcha JT, Wang Y, Ma H, Tai PWL, Gao G (2021). Viral vector platforms within the gene therapy landscape. Signal Transduct Target Ther.

[74] Tian J, Andreadis ST (2009). Independent and high-level dual-gene expression in adult stem-progenitor cells from a single lentiviral vector. Gene Ther.

[75] Yu X, Zhan X, D'Costa J, Tanavde VM, Ye Z, Peng T, Malehorn MT, Yang X, Civin CI, Cheng L (2003). Lentiviral vectors with two independent internal promoters transfer high-level expression of multiple transgenes to human hematopoietic stem-progenitor cells. Mol Ther.

[76] Zhu Y, Feuer G, Day SL, Wrzesinski S, Planelles V (2001). Multigene lentiviral vectors based on differential splicing and translational control. Mol Ther.

[77] Papayannakos C, Daniel R (2013). Understanding lentiviral vector chromatin targeting: working to reduce insertional mutagenic potential for gene therapy. Gene Ther.

[78] Muhuri M, Levy DI, Schulz M, McCarty D, Gao G (2022). Durability of transgene expression after rAAV gene therapy. Mol Ther.

[79] Wang D, Tai PWL, Gao G (2019). Adeno-associated virus vector as a platform for gene therapy delivery. Nat Rev Drug Discov.

[80] Zu H, Gao D (2021). Non-viral vectors in gene therapy: recent development, challenges, and prospects. AAPS J.

[81] Yin H, Kanasty RL, Eltoukhy AA, Vegas AJ, Dorkin JR, Anderson DG (2014). Non-viral vectors for gene-based therapy. Nat Rev Genet.

[82] Kariko K, Muramatsu H, Keller JM, Weissman D (2012). Increased erythropoiesis in mice injected with submicrogram quantities of pseudouridine-containing mRNA encoding erythropoietin. Mol Ther.

[83] Kormann MS, Hasenpusch G, Aneja MK, Nica G, Flemmer AW, Herber-Jonat S, Huppmann M, Mays LE, Illenyi M, Schams A (2011). Expression of therapeutic proteins after delivery of chemically modified mRNA in mice. Nat Biotechnol.

[84] Thompson MG, Burgess JL, Naleway AL, Tyner HL, Yoon SK, Meece J, Olsho LEW, Caban-Martinez AJ, Fowlkes A, Lutrick K (2021). Interim estimates of vaccine effectiveness of BNT162b2 and mRNA-1273 COVID-19 vaccines in preventing SARS-CoV-2 infection among health care personnel, first responders, and other essential and frontline workers-Eight U.S. Locations, December 2020–March 2021. MMWR Morb Mortal Wkly Rep..

[85] Dobrowolski C, Paunovska K, Hatit MZC, Lokugamage MP, Dahlman JE (2021). Therapeutic RNA delivery for COVID and other diseases. Adv Healthc Mater..

[86] Prasad KM, Xu Y, Yang Z, Acton ST, French BA (2011). Robust cardiomyocyte-specific gene expression following systemic injection of AAV: in vivo gene delivery follows a Poisson distribution. Gene Ther.

[87] Cheng Q, Wei T, Farbiak L, Johnson LT, Dilliard SA, Siegwart DJ (2020). Selective organ targeting (SORT) nanoparticles for tissue-specific mRNA delivery and CRISPR-Cas gene editing. Nat Nanotechnol.

[88] HaldAlbertsen C, Kulkarni JA, Witzigmann D, Lind M, Petersson K, Simonsen JB (2022). The role of lipid components in lipid nanoparticles for vaccines and gene therapy. Adv Drug Deliv Rev..

[89] Huang M, Chen Z, Hu S, Jia F, Li Z, Hoyt G, Robbins RC, Kay MA, Wu JC (2009). Novel minicircle vector for gene therapy in murine myocardial infarction. Circulation.

[90] Chen ZY, He CY, Ehrhardt A, Kay MA (2003). Minicircle DNA vectors devoid of bacterial DNA result in persistent and high-level transgene expression in vivo. Mol Ther.

[91] Hu YF, Dawkins JF, Cho HC, Marbán E, Cingolani E (2014). Biological pacemaker created by minimally invasive somatic reprogramming in pigs with complete heart block. Sci Transl Med..

[92] Shlapakova IN, Nearing BD, Lau DH, Boink GJ, Danilo P, Kryukova Y, Robinson RB, Cohen IS, Rosen MR, Verrier RL (2010). Biological pacemakers in canines exhibit positive chronotropic response to emotional arousal. Heart Rhythm.

[93] Dawkins JF, Hu YF, Valle J, Sanchez L, Zheng Y, Marbán E, Cingolani E (2019). Antegrade conduction rescues right ventricular pacing-induced cardiomyopathy in complete heart block. J Am Coll Cardiol.

[94] Huang CY, Liu CL, Ting CY, Chiu YT, Cheng YC, Nicholson MW, Hsieh PCH (2019). Human iPSC banking: barriers and opportunities. J Biomed Sci.

[95] Potapova I, Plotnikov A, Lu Z, Danilo P, Valiunas V, Qu J, Doronin S, Zuckerman J, Shlapakova IN, Gao J (2004). Human mesenchymal stem cells as a gene delivery system to create cardiac pacemakers. Circ Res.

[96] Tse HF, Xue T, Lau CP, Siu CW, Wang K, Zhang QY, Tomaselli GF, Akar FG, Li RA (2006). Bioartificial sinus node constructed via in vivo gene transfer of an engineered pacemaker HCN Channel reduces the dependence on electronic pacemaker in a sick-sinus syndrome model. Circulation.

[97] Shirley JL, de Jong YP, Terhorst C, Herzog RW (2020). Immune responses to viral gene therapy vectors. Mol Ther.

[98] Kim NK, Wolfson D, Fernandez N, Shin M, Cho HC (2019). A rat model of complete atrioventricular block recapitulates clinical indices of bradycardia and provides a platform to test disease-modifying therapies. Sci Rep.

[99] Klug D, Balde M, Pavin D, Hidden-Lucet F, Clementy J, Sadoul N, Rey JL, Lande G, Lazarus A, Victor J (2007). Risk factors related to infections of implanted pacemakers and cardioverter-defibrillators: results of a large prospective study. Circulation.

[100] Baddour LM, Cha YM, Wilson WR (2012). Clinical practice Infections of cardiovascular implantable electronic devices. N Engl J Med..

[101] Sohail MR, Henrikson CA, Braid-Forbes MJ, Forbes KF, Lerner DJ (2011). Mortality and cost associated with cardiovascular implantable electronic device infections. Arch Intern Med.

[102] Boyle TA, Uslan DZ, Prutkin JM, Greenspon AJ, Baddour LM, Danik SB, Tolosana JM, Le K, Miro JM, Peacock J, et al. Reimplantation and repeat infection after cardiac-implantable electronic device infections: experience from the medic (multicenter electrophysiologic device infection cohort) database. Circul Arrhyth Electrophysiol. 2017;10. 10.1161/circep.116.004822. 10.1161/CIRCEP.116.004822 28292753

[103] Abu-El-Haija B, Bhave PD, Campbell DN, Mazur A, Hodgson-Zingman DM, Cotarlan V, Giudici MC (2015). Venous stenosis after transvenous lead placement: a study of outcomes and risk factors in 212 consecutive patients. J Am Heart Assoc.

[104] Morani G, Bolzan B, Valsecchi S, Morosato M, Ribichini FL (2020). Chronic venous obstruction during cardiac device revision: incidence, predictors, and efficacy of percutaneous techniques to overcome the stenosis. Heart Rhythm.

[105] Boveda S, Lenarczyk R, Haugaa KH, Iliodromitis K, Finlay M, Lane D, Prinzen FW, Dagres N (2018). Use of leadless pacemakers in Europe: results of the European Heart Rhythm Association survey. Europace.

[106] van Eck JW, van Hemel NM, Zuithof P, van Asseldonk JP, Voskuil TL, Grobbee DE, Moons KG (2007). Incidence and predictors of in-hospital events after first implantation of pacemakers. Europace.

[107] Armaganijan LV, Toff WD, Nielsen JC, Andersen HR, Connolly SJ, Ellenbogen KA, Healey JS (2012). Are elderly patients at increased risk of complications following pacemaker implantation? A meta-analysis of randomized trials. Pacing Clin Electrophysiol PACE.

[108] Guha A, Maddox WR, Colombo R, Nahman NS, Kintziger KW, Waller JL, Diamond M, Murphy M, Kheda M, Litwin SE (2015). Cardiac implantable electronic device infection in patients with end-stage renal disease. Heart Rhythm.

[109] Bevins MB (2011). The ethics of pacemaker deactivation in terminally ill patients. J Pain Symptom Manage.

[110] Pitcher D, Soar J, Hogg K, Linker N, Chapman S, Beattie JM, Jones S, George R, McComb J, Glancy J (2016). Cardiovascular implanted electronic devices in people towards the end of life, during cardiopulmonary resuscitation and after death: guidance from the Resuscitation Council (UK), British Cardiovascular Society and National Council for Palliative Care. Heart (British Cardiac Society).

[111] Pasalic D, Gazelka HM, Topazian RJ, Buchhalter LC, Ottenberg AL, Webster TL, Swetz KM, Mueller PS (2016). Palliative care consultation and associated end-of-life care after pacemaker or implantable cardioverter-defibrillator deactivation. Am J Hosp Palliat Care.

[112] Hindricks G, Potpara T, Dagres N, Arbelo E, Bax JJ, Blomström-Lundqvist C, Boriani G, Castella M, Dan GA, Dilaveris PE (2021). 2020 ESC Guidelines for the diagnosis and management of atrial fibrillation developed in collaboration with the European Association for Cardio-Thoracic Surgery (EACTS): The Task Force for the diagnosis and management of atrial fibrillation of the European Society of Cardiology (ESC) Developed with the special contribution of the European Heart Rhythm Association (EHRA) of the ESC. Eur Heart J.

[113] Groner A, Grippe K (2019). The leadless pacemaker: an innovative design to enhance pacemaking capabilities. Jaapa.

[114] Bari Z, Vamos M, Bogyi P, Reynolds D, Sheldon T, Fagan DH, Duray GZ (2018). Physical activity detection in patients with intracardiac leadless pacemaker. J Cardiovasc Electrophysiol.

[115] Brito-Zerón P, Izmirly PM, Ramos-Casals M, Buyon JP, Khamashta MA (2015). The clinical spectrum of autoimmune congenital heart block. Nat Rev Rheumatol.

[116] Schmidt KG, Ulmer HE, Silverman NH, Kleinman CS, Copel JA (1991). Perinatal outcome of fetal complete atrioventricular block: a multicenter experience. J Am Coll Cardiol.

[117] Jaeggi ET, Hamilton RM, Silverman ED, Zamora SA, Hornberger LK (2002). Outcome of children with fetal, neonatal or childhood diagnosis of isolated congenital atrioventricular block. A single institution's experience of 30 years. J Am Coll Cardiol..

[118] Baruteau AE, Pass RH, Thambo JB, Behaghel A, Le Pennec S, Perdreau E, Combes N, Liberman L, McLeod CJ (2016). Congenital and childhood atrioventricular blocks: pathophysiology and contemporary management. Eur J Pediatr.

[119] Fortescue EB, Berul CI, Cecchin F, Walsh EP, Triedman JK, Alexander ME (2004). Patient, procedural, and hardware factors associated with pacemaker lead failures in pediatrics and congenital heart disease. Heart Rhythm.

[120] Takeuchi D, Tomizawa Y (2013). Pacing device therapy in infants and children: a review. J Artif Organs.

[121] Zhao H, Yang M, Wang F, Yang A, Zhao Q, Wang X, Tang Y, Wang T, Huang C (2019). Overexpression of the medium-conductance calcium-activated potassium channel (SK4) and the HCN2 channel to generate a biological pacemaker. Mol Med Rep.

[122] Gorabi AM, Hajighasemi S, Khori V, Soleimani M, Rajaei M, Rabbani S, Atashi A, Ghiaseddin A, Saeid AK, Ahmadi Tafti H (2019). Functional biological pacemaker generation by T-Box18 protein expression via stem cell and viral delivery approaches in a murine model of complete heart block. Pharmacol Res.

[123] Plotnikov AN, Bucchi A, Shlapakova I, Danilo P, Brink PR, Robinson RB, Cohen IS, Rosen MR (2008). HCN212-channel biological pacemakers manifesting ventricular tachyarrhythmias are responsive to treatment with I(f) blockade. Heart Rhythm.

